# Using publicly available datasets to identify population-based transcriptomic landscape contributing to the aggressiveness of breast cancer in young women

**DOI:** 10.3389/fgene.2022.1039037

**Published:** 2023-01-04

**Authors:** Marah Tabbal, Mahmood Y. Hachim, Reem Kais Jan, Thomas E. Adrian

**Affiliations:** College of Medicine, Mohammed Bin Rashid University of Medicine and Health Sciences, Dubai Healthcare City, Dubai, United Arab Emirates

**Keywords:** early-onset, breast cancer, aggressive, metastasis, genomics, amplification, transcriptomics, *in-silico*

## Abstract

**Introduction:** Although the risk of breast cancer increases with advancing age, some regions have larger number of young breast cancer patients (≤45 years-old), such as the Middle East, Eastern Asia, and North Africa, with more aggressive and poorly differentiated tumors. We aimed to conduct an *in-silico* analysis in an attempt to understand the aggressive nature of early-onset breast cancer, and to identify potential drivers of early-onset breast cancer using gene expression profiling datasets in a population-dependent manner.

**Methods:** Functional genomics experiments data were acquired from cBioPortal database for cancer genomics, followed by the stratification of patients based on the age at representation of breast cancer and race. Differential gene expression analysis and gene amplification status analysis were carried out, followed by hub gene, transcription factor, and signalling pathway identification.

**Results:** PAM50 subtype analysis revealed that young patients (≤45 years-old) had four-fold more basal tumors and worst progression-free survival (median of 101 months), compared with the 45–65 years group (median of 168 months). Fourteen genes were amplified in more than 14% of patients with an early-onset breast cancer. Interestingly, FREM2, LINC00332, and LINC00366 were exclusively amplified in younger patients. Gene expression data from three different populations (Asian, White, and African) revealed a unique transcriptomic profile of young patients, which was also reflected on the PAM50 subtype analysis. Our data indicates a higher tendency of young African patients to develop basal tumors, while young Asian patients are more prone to developing Luminal A tumors. Most genes that were found to be upregulated in younger patients are involved in important signaling pathways that promote cancer progression and metastasis, such as MAPK pathway, Reelin pathway and the PI3K/Akt pathway.

**Conclusion:** This study provides strong evidence that the molecular profile of tumors derived from young breast cancer patients of different populations is unique and may explain the aggressiveness of these tumors, stressing the need to conduct population- based multi-omic analyses to identify the potential drivers for tumorigenesis and molecular profiles of young breast cancer patients.

## 1 Introduction

**FIGURE 1:**
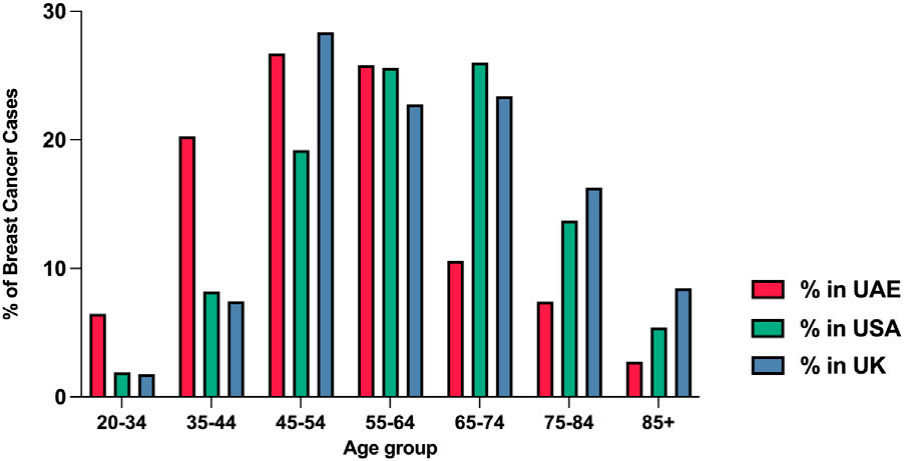
The percentage of breast cancer cases by age groups in the UAE, United States, and United Kingdom in 2017. Data obtained from the annual UAE report-national cancer registry 2017, NIH SEER incidence database, and Office for National Statistics (United Kingdom) (Cancer Incidence, 2017).

As breast cancer remains a global burden that accounts for approximately a quarter of all female cancer cases worldwide, research has been focused on identifying the risk factors and molecular mechanisms underlying females’ susceptibility for developing breast cancer. Despite advances in science and medicine, breast cancer remains a burden, being the most frequently diagnosed cancer amongst women worldwide and the second cause of cancer-related deaths in women (GLOBOCAN, 2020) (Sung et al., 2021). Advancing age is one of the main risk factors for developing breast cancer (Cancer), with only 10% of all breast cancer cases in the United States being diagnosed between 20–44 years of age (Cancer). However, the frequency of breast cancer in premenopausal women (<40 years) is reported to be high in some regions, such as the Middle East, Eastern Asia, and North Africa, where the trend of developing young age breast cancer is going up with time (Najjar and Easson, 2010; Hironaka-Mitsuhashi et al., 2017). Figure 1 displays the age distribution of breast cancer cases amongst United Arab Emirates nationals in 2017 and shows that 41.9% of breast cancer cases were diagnosed before the age of 49 years (Cancer Incidence, 2017).

A meta-analysis by Najjar et al., in 2010 reported the average age of diagnosis of breast cancer in Arab women from 18 countries to be 48 years (SD = 2.8), a decade earlier than their Western counterparts (Najjar and Easson, 2010). Moreover, in 2013 Chouchane et al. reported that breast cancer in the Arab population appears at a younger age, with a different presentation compared to other regions (Chouchane et al., 2013). Breast cancer in premenopausal women is usually accompanied by a higher mortality rate and a worse disease-free survival compared to postmenopausal women (Collins et al., 2012). These tumors are often poorly differentiated, leading to unfavorable outcomes and poor survival in younger women (Cathcart-Rake et al., 2021). Hence, it is crucial to understand the molecular mechanisms underlying the development and progression of aggressive early-onset breast cancer, which can lead to the development of management strategies and potential targeted therapies to tackle these tumors. An essential aspect to consider when studying the molecular mechanisms underlying breast tumors and developing effective targeted treatments is the intertumoral and intratumor heterogeneity (Turashvili and Brogi, 2017). Intratumor heterogeneity is characterized by the presence of diverse cell populations within a single tumor, which makes these tumors more challenging to treat (Ellsworth et al., 2017). In addition, breast tumors differ in many other aspects, including their genomic, transcriptomic, proteomic, and metabolomic profiles, resulting in diagnostic and therapeutic challenges (Perou and Børresen-Dale, 2011).

This study is divided into two sections; the first section is a review of the available literature on molecular and biological aspects that can potentially explain the aggressive nature of breast cancer in young women. In the second section, we adopted an *in silico* approach where we reanalyzed publicly available bioinformatic datasets retrieved from cBioPortal to identify the most common genomic aberrations found in young women with breast cancer. In addition, we analyzed differentially expressed genes (DEGs) between young women (45 years old and below), older women (between 45–65 years old and above 65 years old) with breast cancer, to differentiate between the tumors found in these two sub-populations.

The importance of the investigation of the genomic aspects of breast cancer is inevitable in an era of personalized therapeutics. When it comes to breast cancer genomics, two of the most important genes that can possibly increase the lifetime risk of hereditary breast and ovarian cancer in general, and early-onset breast cancer in specific, are the BRCA1/2 genes (Kuchenbaecker et al., 2017). These genes are mainly involved in the repair of DNA double-stranded breaks by homologous recombination repair (HRR), an essential DNA repair process (Roy et al., 2011). Pathogenic variants and germline mutations in BRCA1 and BRCA2 can increase the risk of developing breast cancer at a younger age, as these mutations represent more than 50% of all mutations in breast cancer-related genes in young women of specific populations (Chelmow et al., 2020). For instance, the age of onset of breast cancer in Japanese patients harboring germline mutations in BRCA1/2 was lower than the age of onset in patients with intact BRCA1/2, with women younger than 40 years of age having basal or basal-like tumors (Okano et al., 2021).

In Brazilian early-onset breast cancer patients (age ≤35 years) cohort, the frequency of germline mutations in BRCA1/2 with luminal tumors is reported to be 16% (Encinas et al., 2018), and 43% of luminal non-BRCA1/2 mutant tumors harbored pathogenic variants in DNA repair genes (ATR, BAP1, ERCC6, FANCD2, FANCL, MLH1, MUTYH, PALB2, POLD1, POLE, RAD9A, RAD51 and TP53), whereas 54% had pathogenic variants in transcription-related genes.

PALB2 is another important gene that plays a role in DNA repair by HRR, as it interacts with BRCA2 and forms a complex that leads to the translocation of RAD51 to the double stranded break for repair (Zhang et al., 2009). PALB2 mutations has been observed to be a risk factor for early-onset breast cancer in several populations (Foulkes et al., 2007; Southey et al., 2010; Teo et al., 2013). For instance, Cecener et al. found PALB2 variants in 4.03% of Turkish young women with breast cancer (Cecener et al., 2016). However, more studies are needed to confirm the importance of PALB2 mutations on the age of onset of breast cancer.

TP53, which is a significant tumor suppressor gene that is mutated in many cancers, was investigated by many research groups for its importance in breast cancer progression (Kaur et al., 2018). McCuaig et al. reported that the frequency of mutations in TP53 amongst women with early-onset breast cancer was 33.3% (McCuaig et al., 2012). In another study that aimed to investigate somatic mutations in tumors from Taiwanese young breast cancer patients, Midha et al. reported a high prevalence of somatic mutations in TP53 (40%) (Midha et al., 2020). This result was concordant with the frequency of TP53 mutations in a pooled premenopausal breast cancer cohort (41.5%), compared to a frequency of 32.9% in pooled postmenopausal breast cancer cohort (Midha et al., 2020).

Additionally, two germline pathogenic variants (rs10963755 and rs715212) were identified in ADAMTSL1 (Kadalayil et al., 2017) that were associated with poor prognosis in young women aged ≤40 years with breast cancer. It was also reported that rs71521 may influence the expression of AREG, known to be involved in the localization of EGFR to the plasma membrane, which eventually leads to cellular invasion and a more aggressive phenotype (Baillo et al., 2011).

Differential expression of genes can play a vital role in the development, aggressiveness, invasiveness, metastasis, and poor prognosis of breast cancer in young women. In a study that assessed age-related differentially expressed genes in breast cancer patients, Johnson et al. analyzed the expression of 17 genes involved in the invasive nature of breast tumors (Johnson et al., 2015). Out of the 17 candidate genes, and after correction for tumor subtype and grade, they found that four genes (BUB1, KRT5, MYCN, and CXCL12) were differentially expressed in young versus older women (<40 years vs. ≥ 40 years) with breast cancer. While BUB1, KRT5, and MYCN were overexpressed in young women with breast cancer, CXCL12 showed a decreased expression. The genes that were overexpressed in young women were associated with high proliferation rate of the breast tumors and with poor prognosis.

A significant overexpression in estrogen-responsive genes, such as GREB1 and AREG, was observed in estrogen-receptor positive (ER+) breast cancer patients aged 45 years or younger, who had a significantly lower expression of ESR1 compared to older women (age ≥70 years) (Yau et al., 2007). In another study, it was also reported that older women had an overexpression of ESR1 compared to younger women (Eppenberger-Castori et al., 2002). This was accompanied by an overexpression of growth factor receptors (ErbB2 or EGFR) in the younger group, making receptor-positive tumors in young women more likely to be aggressive with a higher proliferation rate.

Another important gene that was found to be overexpressed in young women is FOXM1, as its’ overexpression was associated with poor outcomes in ER + young breast cancer patients compared to older patients (Yau et al., 2011). In addition, it was reported that ER + tumors in young patients had a higher proliferative ability and the women experienced more metastatic events compared to older women with ER-positive tumors.

Postpartum young women aged 45 years or younger with ER + breast cancer showed increased upregulation of transcription factors associated with cell cycle progression (E2F1 and E2F4), and downregulation of TP53 and ESR1, when compared with nulliparous women. Although all patients enrolled were clinically ER+, differentially expressed regulons between postpartum and nulliparous young breast cancer patients were transcription factors that are differentially expressed between ER- and ER + breast tumors. Unsupervised clustering based on PAM50 gene expression values showed that luminal A postpartum young patients clustered with poor prognosis luminal B nulliparous patients, which indicates that luminal A postpartum patients have poorer outcomes compared to luminal A nulliparous patients (Jindal et al., 2021).

Based on this data, the expression profiles of young women show a great potential for understanding the aggressive nature of early-onset breast cancer. However, more studies are needed to identify population-based expression markers that can potentially help in developing targeted therapeutics for these patients.

Analyzing the breast cancer proteome and its association with poor outcomes of early-onset breast cancer patients is vital for the accurate diagnosis and therapeutic decision, which can prolong the overall survival of those patients. However, only few studies investigated the differentially expressed proteins in early-versus late-onset breast cancer patients. Bastos et al. identified seven proteins that were differentially expressed in young (≤35 years) versus middle aged (50–65 years) breast cancer patients (Bastos et al., 2016). Three of these proteins (BCL2L1, PARP1, and RAF1) were upregulated in younger patients, whereas four proteins (ESR1, EIF4E, STAT5A, and RPS6KA1) were under expressed in these younger patients. While the overexpression of BCL2L1 is known to be associated with anti-apoptotic functions, PARP1 is known for its importance in DNA repair by non-homologous end joining (Wang et al., 2017; Warren et al., 2019). In addition, the overexpression of RAF1 is linked to a more aggressive phenotype of breast cancer, leading to poor outcomes and prognosis in patients (Wu et al., 2014). These results support the idea that early breast cancer is more aggressive and has a higher metastatic potential compared to breast cancer affecting older women.

Jordan et al. analyzed the proteomic profile of extracellular vesicles extracted from tumors of premenopausal patients with breast cancer and identified a unique proteomic profile in these extracellular vesicles (EV) (Jordan et al., 2020). By analyzing the proteomic content of EVs extracted from young patients with breast cancers matched with samples of healthy donors, they detected increased levels of Mucin 5b, Mucin 1, TIMP1, Laminin B1, Latent TGFB binding protein1, and Myc target proteins. Further analysis of the functionality of this dysregulation and content of EVs identified aberrant roles of genes related to cell motility, progression through the cell cycle, angiogenesis, and epithelial-to-mesenchymal transition, which are characteristics of metastatic tumors. Further analysis using the KEGG database revealed an EV-induced alteration of the focal adhesion kinase pathway, which acts as a mediator of cell invasion and migration (Almstedt et al., 2017).

Identifying signalling pathways that are affected in young breast cancer patients’ tumors can be a powerful tool that could potentially lead to the development of targeted therapies and predicting the prognosis of young women with breast cancer. Riaz et al. identified an alteration in the hedgehog pathway of young women (<45 years old) with breast cancer (Riaz et al., 2018). The upregulation of sonic hedgehog (SHH), desert hedgehog (DHH), and glioma-associated oncogene homolog-1 (GLI-1) was reported to be associated with a higher metastatic ability of these tumors. The overexpression of SHH is important for idealizing the tumor microenvironment for metastasis and progression, whereas the overexpression of GLI1 leads to the overexpression of matrix metalloprotease-1 (MMP-1) and vascular endothelial growth factor A (VEGFA), increasing the tumors’ metastatic potential (O’Toole et al., 2011; Kuehn et al., 2021).

Colak et al. assessed the differentially expressed genes associated with signaling pathways involved in carcinogenesis and metastasis in young Middle Eastern women (<45 years) (Colak et al., 2013). The genes that were differentially expressed between young and older women were mainly involved in the regulation of PI3K/Akt, MYC, and NF-κB signalling pathways. Aberrations in the PI3K/Akt pathway are linked to resistance to endocrine therapy in hormone-receptor positive breast cancer, and resistance to trastuzumab in HER-2 positive tumors (Paplomata and O’Regan, 2014; Nunnery and Mayer, 2020). On the other hand, the dysregulation of MYC facilitates angiogenesis and metastasis, leading to poor prognosis in breast cancer patients (Baudino et al., 2002; Ma et al., 2010). The NF-κB pathway is a major player in the progression and proliferation of breast cancer tumors, as it promotes epithelial-to-mesenchymal transition (Wang et al., 2014). The findings above can potentially explain the aggressive nature and chemoresistance of early-onset breast cancer. Therefore, the identification of aberrant signaling pathways may provide a valuable therapeutic strategy for treating and managing breast cancer in young women.

## 2 Bioinformatic analysis

### 2.1 Aims of the study

• To conduct an *in silico* analysis in an attempt to understand the aggressive nature of early-onset breast cancer
• To identify potential drivers of early-onset breast cancer in different populations using gene expression profiling datasets and further analyze the data

### 2.2 Materials and methods

**FIGURE 2:**
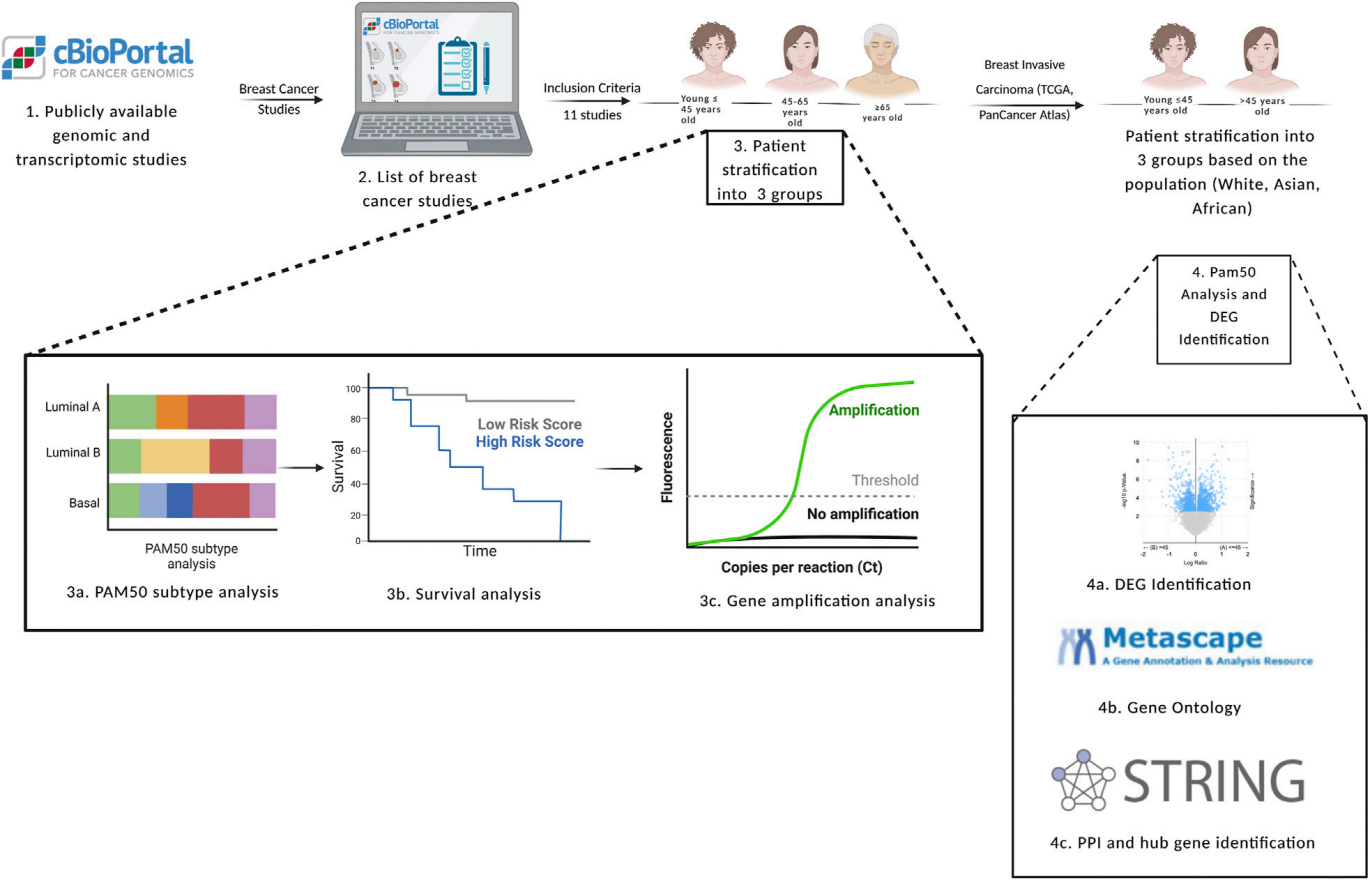
Flowchart diagram of the inclusion and exclusion criteria used in the study, in addition to the methods used to achieve the aims of the study. DEG: differentially expressed genes, PAM50: prediction analysis of microarray 50. Created with BioRender.com.

Figure 2 represents a flowchart diagram of the approaches used to satisfy the aims of the study.

#### 2.2.1 Inclusion criteria

Patients who were included in this study were women who are diagnosed with invasive ductal carcinoma, invasive lobular carcinoma, invasive breast carcinoma, and breast mixed lobular and ductal carcinoma with a reported age at diagnosis. For the differential gene expression analysis, women from white, Asian, and African populations were included in our study. Only women who did not have a reported age at diagnosis were excluded.

#### 2.2.2 Data acquisition

Functional genomics experiments were acquired from cBioPortal for cancer genomics (https://www.cbioportal.org). Twenty-one cBioPortal breast cancer studies representing 10,518 samples were identified, of which only 11 studies satisfied the inclusion criteria of the study (7,357 patients) (Table 1). Patients with invasive ductal carcinoma, invasive lobular carcinoma, invasive breast carcinoma, and breast mixed lobular and ductal carcinoma were selected (7,007 patients). Patient overlapping in different studies were excluded from the analysis, as well as patients with no reported age at diagnosis (3,579 patients). Samples that fulfilled the inclusion criteria (3,428 patients) were further analyzed using the cBioPortal functions. Patients were grouped into three groups based on their age at diagnosis: young patients ≤45 years, middle-aged patients between 45 and 65 years, and patients older than 65 years.

**TABLE 1:** List of studies that satisfied the inclusion criteria and were used for gene amplification analysis.

Names of the studies included in the analysis
Metastatic Breast Cancer (MSK, Cancer Discovery 2021)
Breast Cancer (METABRIC, Nature 2012 & Nat Commun 2016)
Breast Cancer (SMC 2018)
Breast Invasive Carcinoma (British Columbia, Nature 2012)
Breast Invasive Carcinoma (Broad, Nature 2012)
Breast Invasive Carcinoma (Sanger, Nature 2012)
Breast Invasive Carcinoma (TCGA, Firehose Legacy)
Breast Invasive Carcinoma (TCGA, PanCancer Atlas)
Breast Invasive Carcinoma (TCGA, Nature 2012)
MAPK on resistance to anti-HER2 therapy for breast cancer (MSKCC, Nat Comm 2021)
Proteogenomic landscape of breast cancer (CPTAC, Cell 2020)

#### 2.2.3 PAM50 cancer type analysis

For the selected cohort of patients, three groups were defined based on their age: ≤45 years old, 45–65 years old, and >65 years old. The number of patients below the age of 45 that were included in the analysis was 763 patients, whereas the number of patients between 45–65 years old was 1779 patients, and 886 patients were above the age of 65 years old. PAM50 cancer type analysis and a survival analysis were done using the clinical function in cBioPortal.

#### 2.2.4 Gene amplification analysis

After correlating age at diagnosis with gene amplification status, genes that were amplified in the younger patients with a *p* < 0.05 were identified. Genes that were considered for further analysis in our study had a minimum of 14% alteration event frequency in the younger age group.

#### 2.2.5 Population based differential gene expression analysis

Differentially expressed genes were analyzed from a single study of 1,084 patients from three different populations (Breast Invasive Carcinoma (TCGA, PanCancer Atlas). Patients were stratified based on the population of origin (Asian, African, and White), then they were sub-stratified by age into two groups: patients ≤45 years old, and patients >45 years old. Our subgroups included 751 white patients, 182 patients from African origins, and 60 Asian patients. 91 patients were excluded from our study due to unreported race. After correlating selected clinical features with gene expression status, 10,382 genes were found to be differentially expressed between young and older patients of the white population, 9,881 genes in the African population, and 1889 in the Asian population. *p* values were determined using student’s *t*-test analysis, and a *p* < 0.05 was considered a cut-off to include genes that are statistically significant. Genes of log2 value of >1 between young and old patients were considered in our study. mRNA sequencing was done using Illumina HiSeq 2500, and data was normalized using Illumina HiSeq_RNASeqV2. Selected genes were further analyzed for their roles in carcinogenesis from publicly available literature.

#### 2.2.6 Protein-protein interaction network and hub gene identification

Common differentially expressed genes in both datasets were imported to string database (https://string-db.org) to construct a protein-protein interaction (PPI) network. PPI enrichment value is 0.000298, with an average local clustering coefficient of 0.437. The top 10 genes were calculated using CytoHubba, by which the top 10 nodes were ranked by degree and identified as hub genes.

#### 2.2.7 Transcription factor and signaling pathway identification

The list of differentially expressed genes from the Breast Cancer (Breast Invasive Carcinoma (TCGA, PanCancer Atlas) were further annotated on Metascape (https://metascape.org/) to identify the common transcription factors regulating these genes. To identify the signaling pathways that these genes are involved in, genes were enriched and analyzed at Enrichr (https://maayanlab.cloud/Enrichr/). KEGG analysis for signaling pathways was conducted using Enrichr, by which DEGs were sorted according to their *p*-value.

#### 2.2.8 Statistical analysis

Statistical analysis was performed using Prism v9.3.1. Data is represented as mean ± SEM. Statistical significance was evaluated using chi-squared analysis for the gene amplification status analysis (*p* < 0.05). For the differentially expressed genes analysis, statistical significance was evaluated using unpaired student’s *t*-test (*p* < 0.05).

## 3 Results

### 3.1 PAM50 subtype analysis reveals a high proportion of young patients harboring basal tumors

**FIGURE 3:**
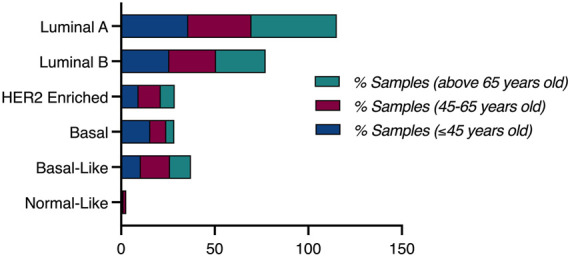
Clinical plot of PAM50 breast cancer subtypes.

Based on the PAM50 cancer type analysis, luminal A breast cancer was prominent in women older than 65 years-old, with 45.68% of those patients harboring luminal A tumors, compared to a frequency of 35.87% in young patients (≤45 years-old), and 33.71% in middle aged patients. However, more than 50% of patients with basal tumors are young patients, as 15.61% of young breast cancer patients (≤45 years-old) had basal tumors, compared to 8.5% of middle-aged patients (45–65 years-old) harbouring basal tumors, and only 4.32% of old women (>65 years-old) had basal tumors (Figure 3).

### 3.2 Kaplan-Meier survival analysis revealed poor progression-free survival in young breast cancer patients

**FIGURE 4:**
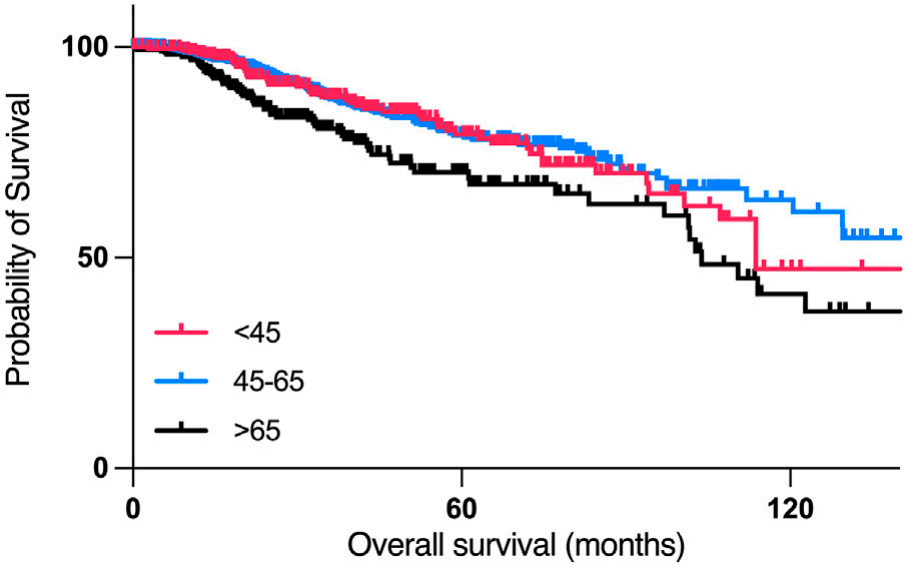
Overall survival analysis of women with breast cancer of different age groups.

According to the Kaplan-Meier survival analysis, patients who are 45 years old or younger had poorer overall survival than patients between 45–65 years old, with a median month survival of 114 months, and 212 months, respectively, with the worst overall survival in the >65 years-old age group, with a median month survival of 103.78 (Figure 4).

**FIGURE 5:**
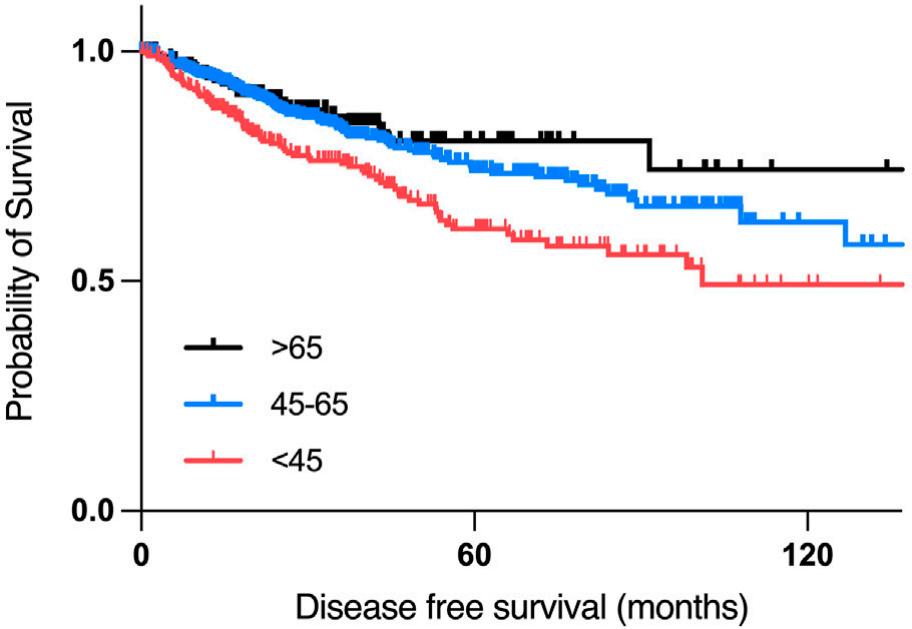
Disease free survival analysis of women with breast cancer of different age groups.

According to the disease-free survival analysis, patients who are aged 45 years or younger had the worst disease-free survival compared to other two age groups, with a median disease-free period of 101.05 months, compared to a median of 168.1 months in the 45–65 years group. (Figure 5).

### 3.3 Fourteen genes are amplified in more than 14% of young breast cancer patients

**FIGURE 6:**
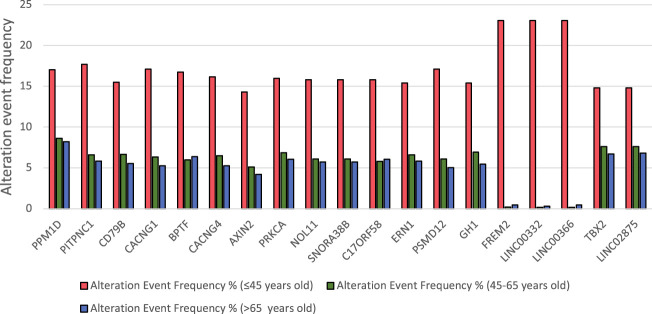
Amplified genes in women with breast cancer of different age groups.

After correlating selected clinical features with gene amplification status, 4500 genes were found to be significantly amplified in the selected cohort of patients (*p* < 0.05, derived from chi-squared analysis). After selecting the genomic alterations that were enriched in women aged 45 years or younger, 2972 genes were found to be significantly amplified in younger women (*p* < 0.05). Genes that were amplified in more than 14% of patients were considered in the study (Figure 6). Genes that were significantly amplified in young patients with breast cancer were further analyzed from published literature for their significance in the aggressiveness of early-onset breast cancer (Table 2).

**TABLE 2:** Significantly amplified genes in women with early onset breast cancer and their clinical significance.

Gene	Genomic location	Significance
PPM1D (Protein Phosphatase 1D)	chr17q23.2	Amplification is associated with the overexpression of ErbB2, promotes cell proliferation and inhibits apoptosis (Rauta et al., 2006)
PITPNC1 (Phosphatidylinositol Transfer Protein Cytoplasmic 1)	chr17q24.2	Amplification is associated with breast cancer metastasis and metastatic progression (Halberg et al., 2016)
CD79B	chr17q23.3	Found to be highly expressed in metastatic lymph node tumors (Ellsworth et al., 2008)
CACNG1 (Calcium Voltage-Gated Channel Auxiliary Subunit Gamma 1)	chr17q24.2	Amplification alters cell motility and promotes metastasis (Kanwar et al., 2020)
BPTF (Bromodomain PHD Finger Transcription Factor)	chr17q24.2	Transcription factor that plays a role in chromatin remodeling and its amplification is associated with metastasis, epithelial-to-mesenchymal transition, and cell migration (Koedoot et al., 2019)
CACNG4 (Calcium Voltage-Gated Channel Auxiliary Subunit Gamma 4)	chr17q24.2	Amplification was reported to alter breast cancer cell motility, induce metastasis, and induce the transformation of benign breast tumors (Kanwar et al., 2020)
AXIN2 (Axis Inhibition Protein 2)	chr17q24.1	Regulates the Wnt/B-catenin pathway, and reported to be more amplified in younger breast cancer patients (Azim et al., 2015)
PRKCA (Protein Kinase C Alpha).	chr17q24.2	Enhances cell migration and promotes metastasis (Pham et al., 2017)
NOL11 (Nucleolar Protein 11)	chr17q24.2	Amplification promotes mitotic entry by the activation of cyclin-dependent kinase 1 (CDK1) (Hayashi et al., 2018)
SNORA38B (Small Nucleolar RNA, H/ACA Box 38B)	chr17q24.2	Small nucleolar RNA that is found in the intronic region of its host gene, NOL11
C17ORF58 (Chromosome 17 Open Reading Frame 58)	chr17p13.1	Reported to up-regulate CENPF gene, which is associated with bone metastasis by the activation of the PI3K-AKT pathway (Sun et al., 2019; Choudhari et al., 2021)
ERN1 (Endoplasmic Reticulum To Nucleus Signaling 1)	chr17q23.3	Understudied kinase, codes for IRE1β, which is an unfolded protein that is part of a cancer pathway hijacked by cancer cells (Essegian et al., 2020)
PSMD12 (Proteasome 26S Subunit, Non-ATPase 12)	crh17q24.2	Promotes cell proliferation and metastasis and inhibits pro-apoptotic genes (Du et al., 2020)
FREM2 (FRAS1 Related Extracellular Matrix 2)	chr13q13.3	Overexpression correlates with poor outcomes in glioma patients (Zolotovskaia et al., 2021)
LINC00332	chr12q13.3	Highest expression in testis (from GTEx RNA-seq of 17,382 samples).
LINC00366	chr6p21.31	Exclusively expressed in male testis (from GTEx RNA-seq of 17,382 samples)
TBX2 (T-Box Transcription Factor 2)	chr17q23.2	The amplification of this gene correlates with the metastatic ability of breast cancer cells through the activation of epithelial-mesenchymal transition and ERK/MAPK signaling pathway (Liu et al., 2019)
LINC02875	chr17q23.2	Upregulation correlates with poor outcomes in patients with glioma (Cheng et al., 2021a)

### 3.4 Most of the amplified genes in young patients are located on chromosome 17q

Amongst 19 genes that were found to be significantly amplified in more than 14% of young patients, 15 genes were found to be located on chromosome 17q. LINC02875, TBX2, and PPM1D were found to be located on chr17q23.2, whereas ERN1 and CD79B were located on chr17q23.3: PITPNC1,CACNG1,BPTF, CACNG1, CACNG4, PRKCA, NOL11, PSMD12 and SNORA38B were found to be located on chr17q24.2.

### 3.5 PAM50 subtype analysis of different populations revealed the highest proportion of basal tumor in Young African patients

**FIGURE 7:**
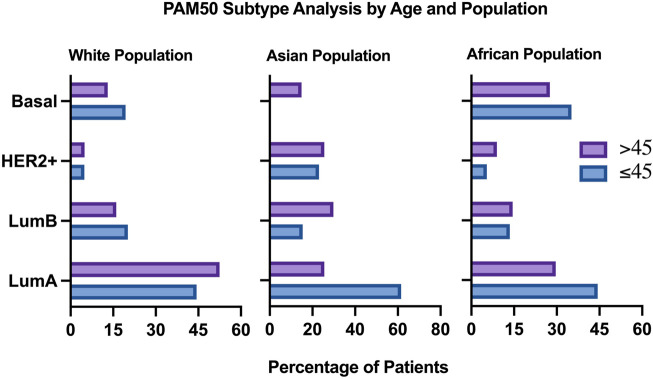
Clinical plot of PAM50 breast cancer subtypes in two different age groups, All populations.

To conduct population specific analysis and identify differentially expressed genes in young women of different populations, we have used the publicly available data of one dataset (TCGA, PanCancer Atlas). Patients were stratified by age, then sub-stratified by race into three populations (African, White, Asian). 751 patients were included in the analysis from the White population, of which 124 (16.5% of patients) were ≤45 years old. PAM50 subtype analysis revealed a higher proportion of young patients having Luminal B and Basal tumors, compared to the older patients who had a higher proportion of Luminal A tumors. The percentage of patients having HER2+ tumors was identical in both age groups of the white population (Figure 7).

In the Asian population, 60 patients were included in the analysis, of which 13 patients (21.7%) were ≤45 years old. Pam50 subtype analysis in this population revealed that patients aged 45 and below most commonly have Luminal A tumors, compared to the older age group. HER2+ and Luminal B tumors were more common in older patients compared to younger patients of this group. Interestingly, none of the young patients in this population had basal tumors, compared to 14.89% of the older population having basal tumors.

In the African population, 182 patients were included in the analysis, by which 37 (21.3%) of them were ≤45 years old. Compared to the previous populations, Pam50 subtype analysis in this population revealed a very high proportion of young African women having Basal and Luminal A tumors, compared to older patients who had a higher proportion of Luminal B and HER2+ patients (Figure 7).

**FIGURE 8:**
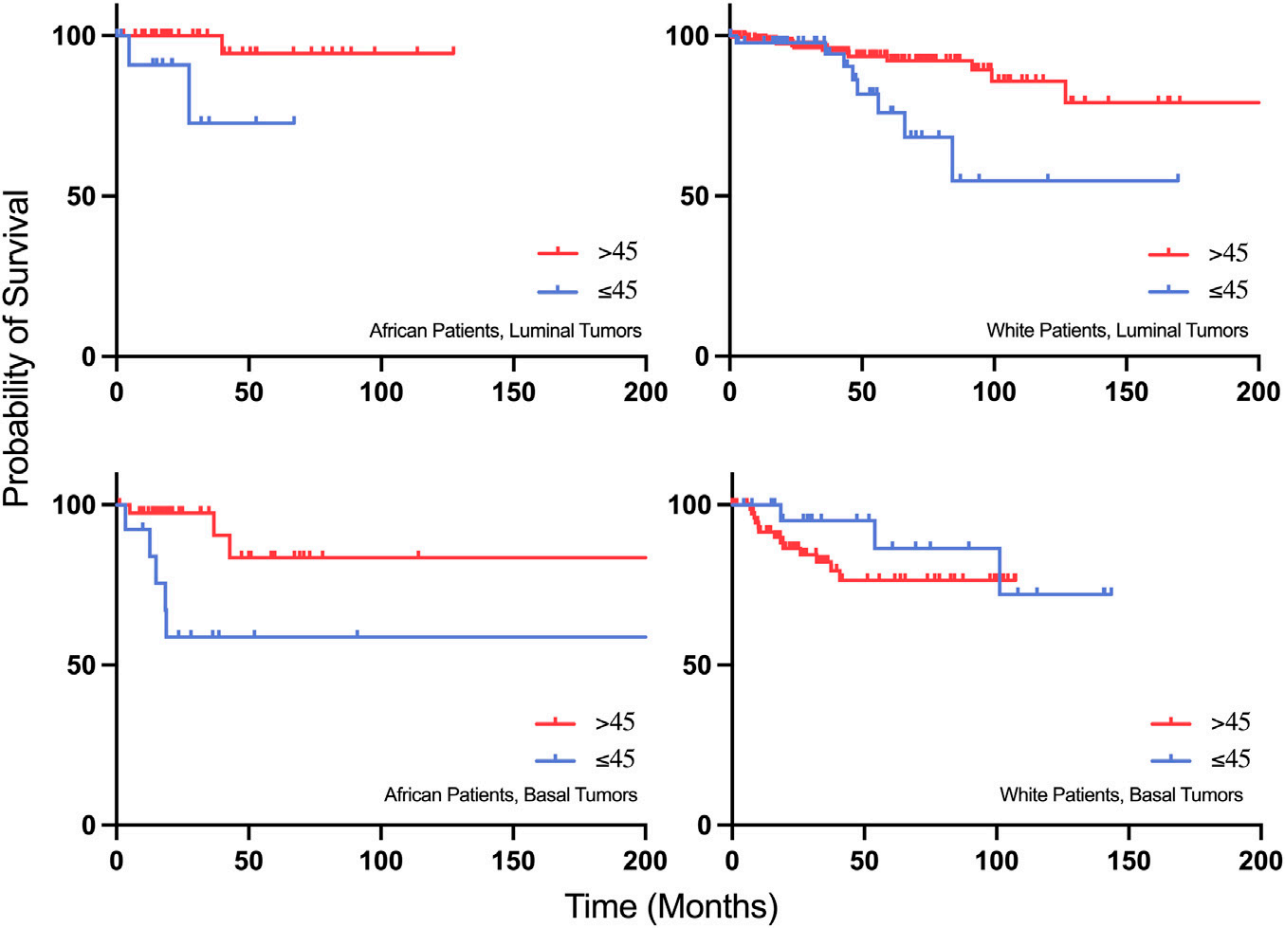
Progression-free survival plots of young African patients with Luminal and Basal tumors compared to young White patients with Luminal and Basal tumors.

Kaplan Meier survival analysis revealed that young African patients (≤45 years old) with luminal and basal tumors have a worse progression free survival compared to older patients (Figure 8). Interestingly, when compared to the white population, young white patients with luminal tumors have a worse progression free survival compared to older patients with the same subtype of cancer. However, young white patients with basal tumors have a better progression free survival compared to the older population (Figure 8).

### 3.6 Differential gene expression analysis revealed a population dependent expression pattern in young breast cancer patients

After correlating selected clinical features with gene expression status in the Breast invasive carcinoma, (TCGA, PanCancer Atlas) cohort, 1,496 genes were significantly overexpressed in young breast cancer patients (≤45 years old) than in the older group (>45 years old). Patients were stratified into three groups based on their race (African, Asian, and White), and genes that were significantly overexpressed and had a log ratio of more than one were selected for further analysis. Genes that were significantly overexpressed in young patients with breast cancer were further analyzed from published literature for their significance in the aggressiveness of early onset breast cancer.

**FIGURE 9:**
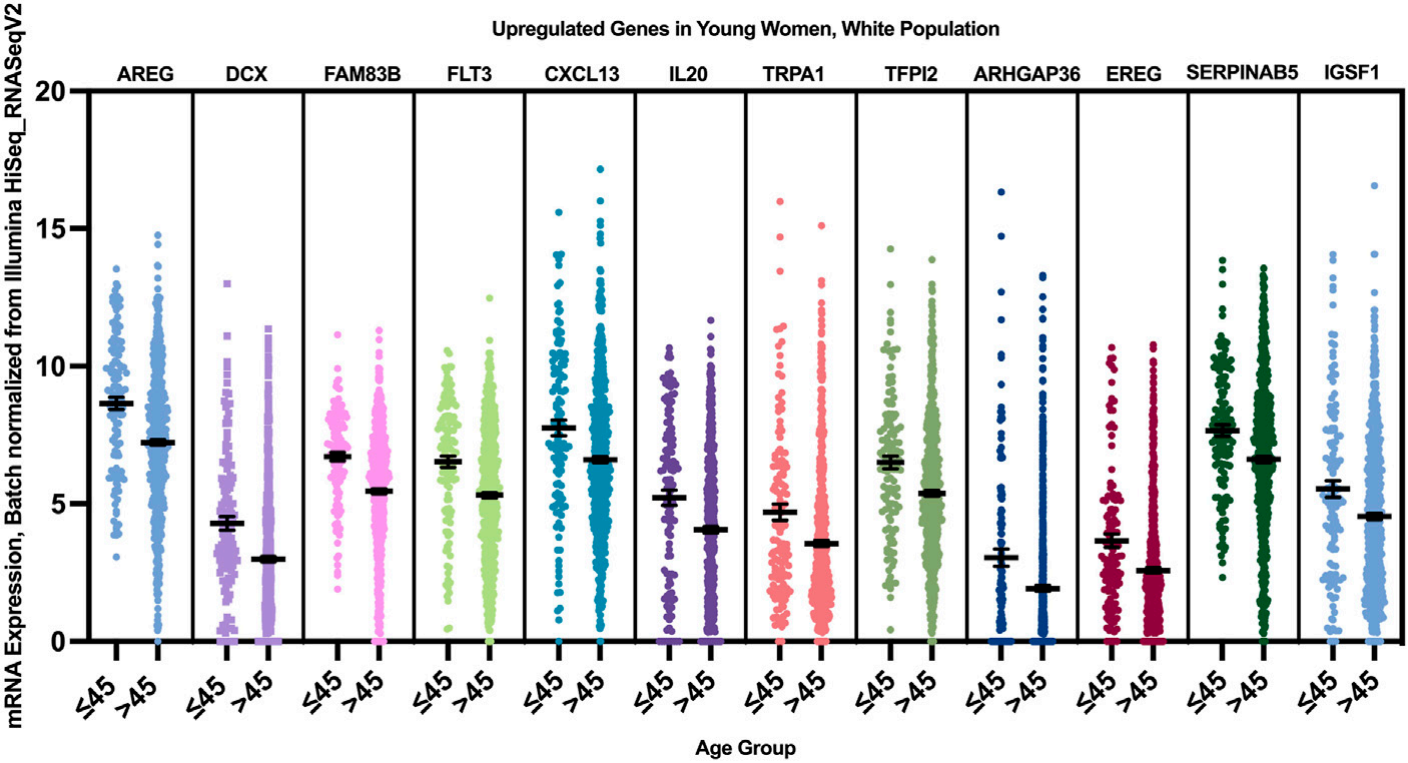
Differentially expressed genes in young vs. older patients of the white population (*p* < 0.005, student’s t-test). Error bars represent Mean ± SEM.

**FIGURE 10:**
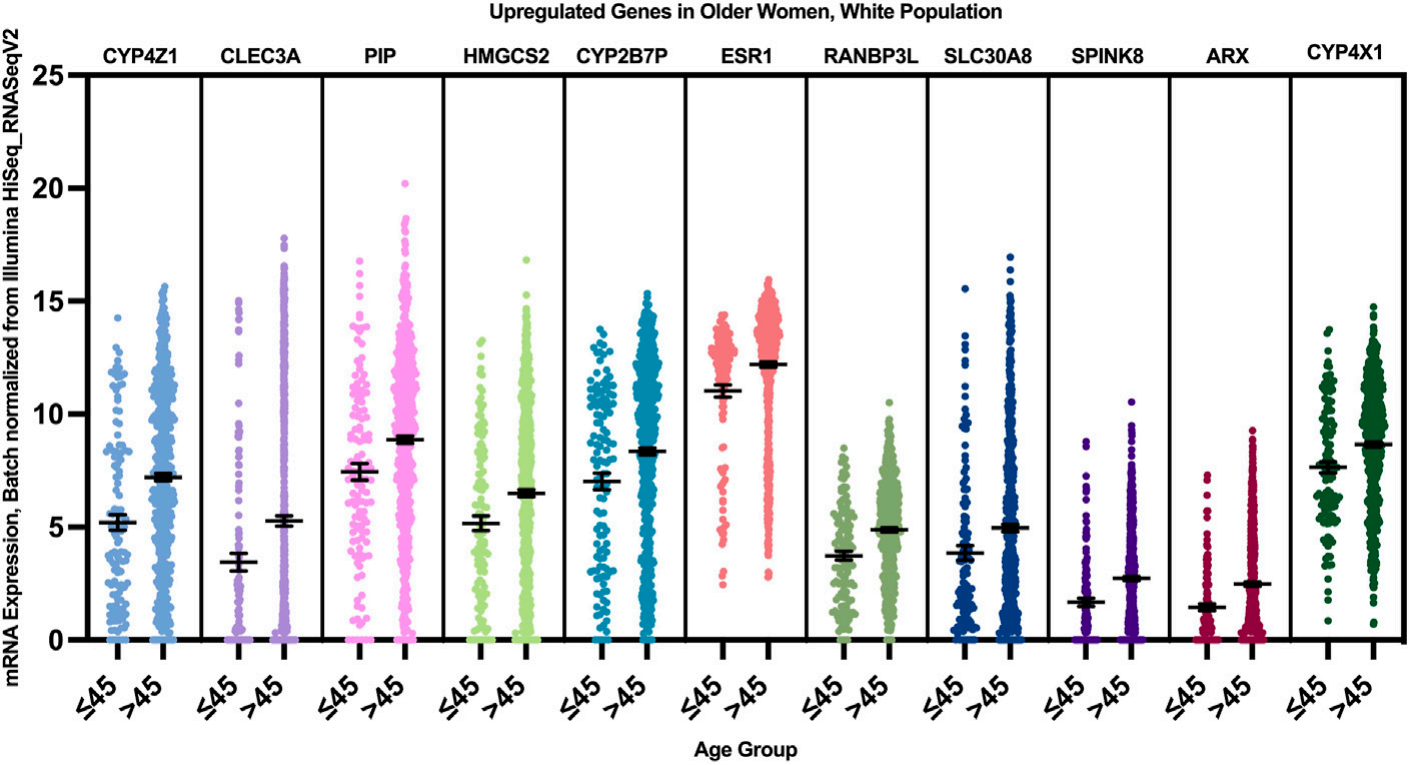
Differentially expressed genes in older vs. young patients of the white population (*p* < 0.005, student’s t-test). Error bars represent Mean ± SEM.

12 genes were found to have a statistically significant higher expression in younger patients of the white population (Figure 9), and 12 genes had a statistically significant higher expression in older patients of the white population (Figure 10).

When we correlated the expression of these genes to the PAM50 subtype they are mainly enriched in, AREG, EREG, and DCX genes were overexpressed in younger women of Luminal A, HER2+ and Luminal B tumors, with a log fold change of >1 compared to older patients. FLT3, CXCL13, IL20, TRPA1, TFPI2, ARHGAP13, and IGSF1 where overexpressed in young women with luminal A and B tumors, while FAM83B was enriched in Luminal A tumors with the highest log fold change compared to older women with luminal tumors, and SERPINAB5 was enriched in young patients with Luminal B tumors with the highest log fold change.

**FIGURE 11:**
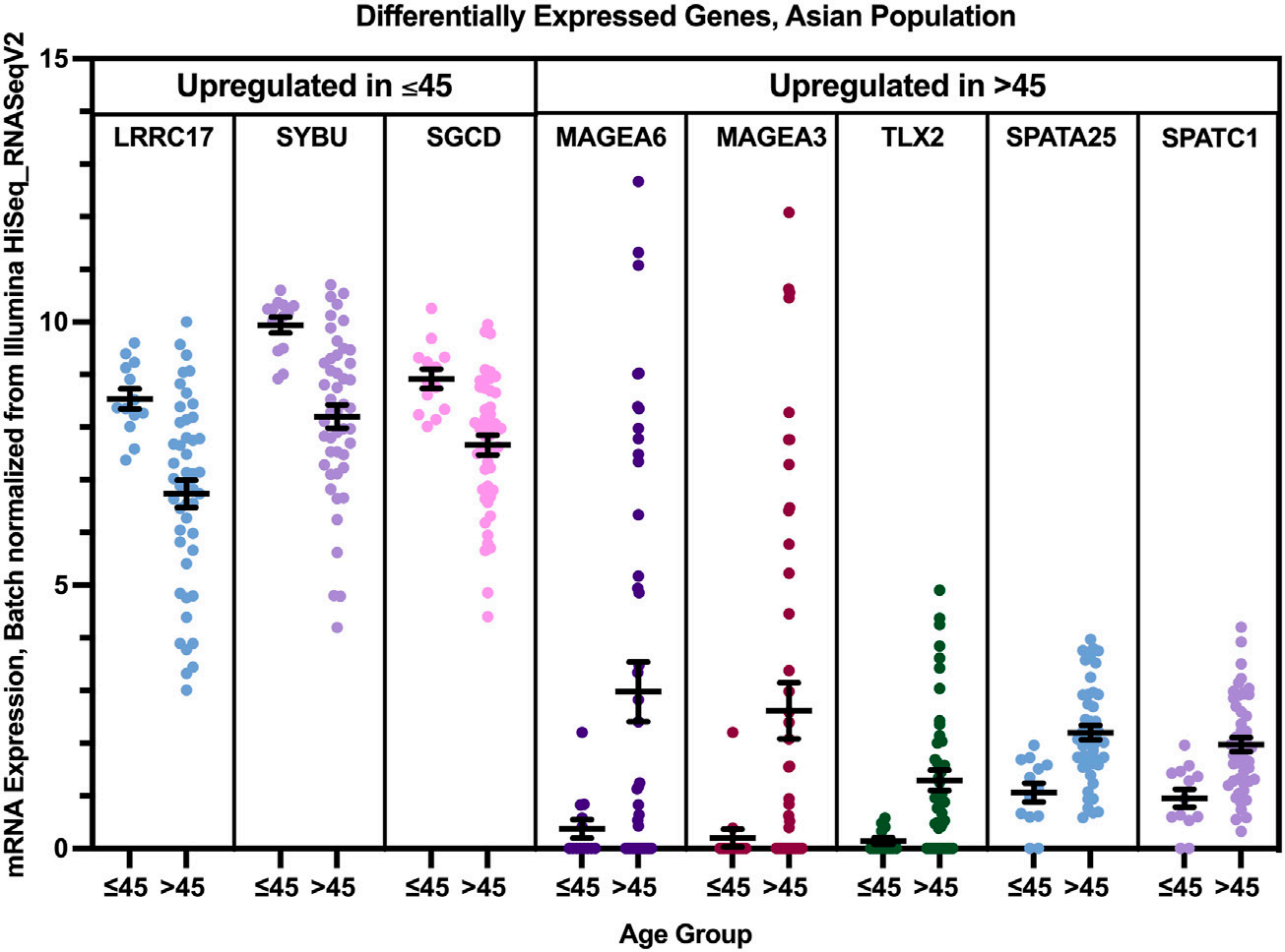
Differentially expressed genes in older and young patients of the Asian population (*p* < 0.005, student’s t-test). Error bars represent Mean ± SEM.

Three genes were found to have a statistically significant higher expression in younger patients of the Asian population, and five genes had a statistically significant higher expression in older patients of the white population (Figure 11). While SGCD and LRRC17 were most significantly overexpressed in young Asian patients with Luminal B and HER2+ compared to older patients with the same tumor types with a log fold change >1, SYBU was found to be overexpressed most significantly in the young patients with HER2+ only, with a log fold change >1, compared to older patients of the same tumor type.

**FIGURE 12:**
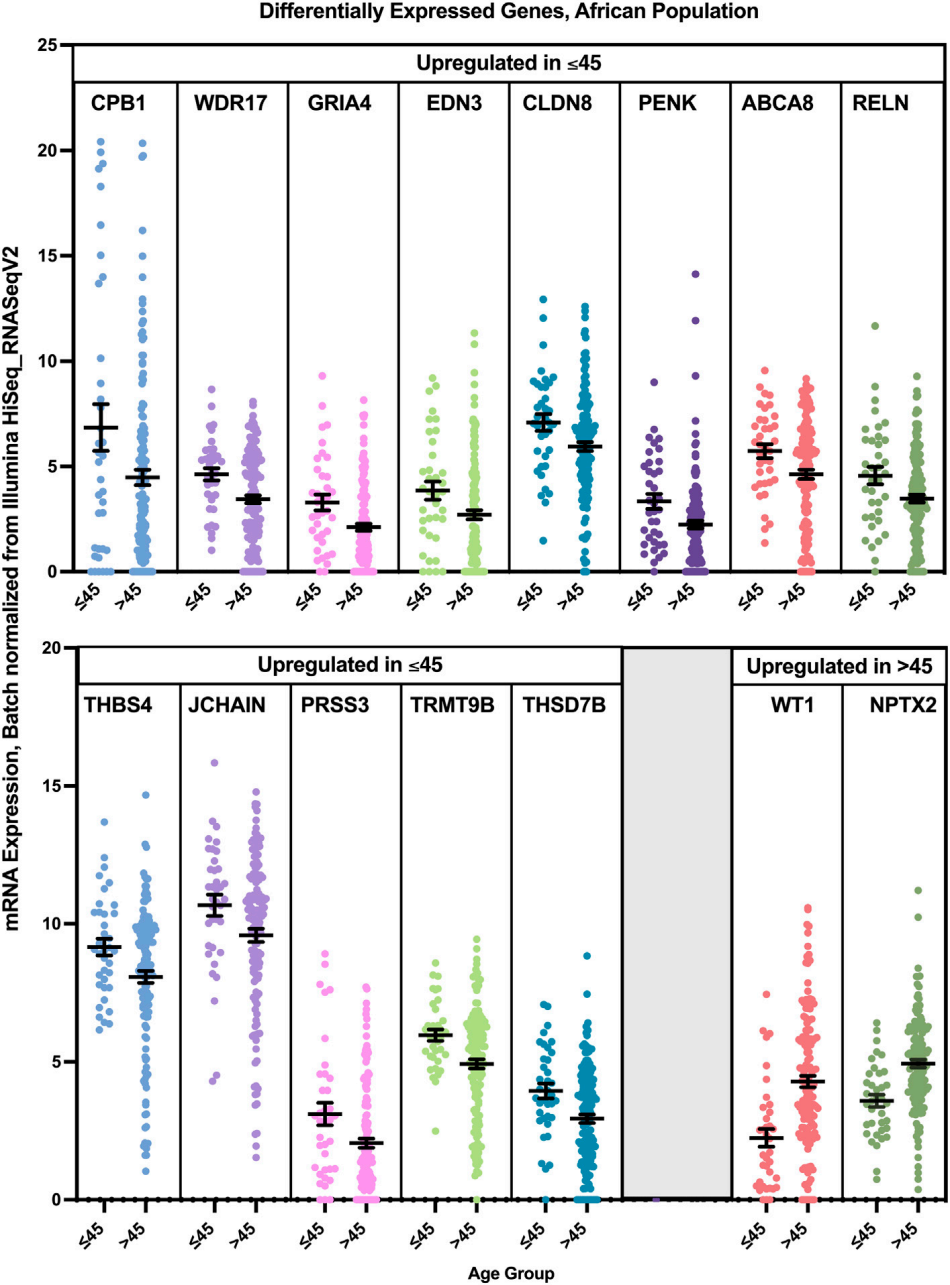
Differentially expressed genes in older and young patients of the Asian population (*p* < 0.05, student’s t-test). Error bars represent Mean ± SEM.

Differential gene expression analysis revealed that 13 genes were found to have a statistically significant higher expression in younger patients of the African population, and two genes had a statistically significant higher expression in older patients of the African population (Figure 12). While PRSS3 was significantly and exclusively enriched in Luminal B tumors of the younger African population, CPB1, ABCA8, JCHAIN, and WDR17 had the highest log fold change mainly in Luminal B and Luminal A tumors of the young patients group. GRIA4 had the highest log fold change in young African patients with Luminal and HER2+ tumors, compared to older patients of the same pam50 subtype, while PENK and RELN where mainly enriched in younger patients with Luminal B and Basal tumors. THSD7B was enriched in younger patients with Luminal and Basal tumors, while the expression of CLDN8 was not subtype specific.

Genes that were found to have a higher expression in younger patients of each population were further analyzed from published literature for their significance in the aggressiveness of early onset breast cancer (Table 3). A follow-up analysis was done to identify the protein-protein interaction (PPI) network, followed by hub gene identification, and pathway analysis.

**TABLE 3:** Differentially expressed genes and their significant roles in carcinogenesis.

DEGs in younger women of the white population	
DEGs	Significance
AREG (Amphiregulin)	EGFR ligand that is involved in the proliferation of breast cancer cells that are ER+ (Peterson et al., 2015)
DCX (Doublecortin)	Overexpressed in migrating neuroblasts, overexpression in cancer could lead to cancer metastasis and migration (Ayanlaja et al., 2017)
FAM83B (Family With Sequence Similarity 83 Member B)	Oncogene and a novel regulator of epithelial-mesenchymal transition in triple negative breast cancer (Bartel et al., 2017)
FLT3 (Fms Related Receptor Tyrosine Kinase 3)	Receptor tyrosine kinase. High expression is reported to have a positive association with favorable outcomes and immune infiltration (Chen et al., 2022)
CXCL13 (C-X-C Motif Chemokine Ligand 13)	High expression correlates with favorable outcomes in breast cancer patients with luminal tumors (Razis et al., 2020)
IL20 (Interleukin 20)	Overexpression is associated with poor outcomes and metastasis in breast cancer patients (Hsu et al., 2012)
TRPA1 (Transient Receptor Potential Ankyrin 1)	Overexpressed in luminal tumors, associated with the upregulation of Ca2+ dependent anti apoptotic pathway, promoting ROS resistance (Takahashi et al., 2018)
TFPI2 (Tissue Factor Pathway Inhibitor 2)	Overexpression suppresses the progression of breast carcinoma (Zhao et al., 2020)
ARHGAP36 (Rho GTPase Activating Protein 36)	Overexpression promotes proliferation and migration of breast cancer cells (Li et al., 2021)
EREG (Epiregulin)	Overexpression mediates breast cancer metastasis to the lungs (Cheng et al., 2021b)
SERPINB5 (Serpin Family B Member 5)	Overexpression correlates with cell motility and cancer metastasis (Vecchi et al., 2007)
IGSF1 (Immunoglobulin Superfamily Member 1)	Inhibition of this gene in thyroid cancer led to the downregulation of N-cadherin, vimentin, and EZH2, which are metastasis associated genes (Guan et al., 2019)
DEGs in Younger Women of the Asian Population	
LRRC17 (Leucine Rich Repeat Containing 17)	Linked to prognosis of ovarian cancer by a TP53 dependent anti-apoptotic pathway (Liu et al., 2021a)
SYBU (Syntabulin)	Microtubule protein. Expression is detected in migrating neuronal cells and metastatic breast cancer cells (Meißner et al., 2017)
SGCD (Sarcoglycan, Delta)	Forms a link between the F-actin cytoskeleton and the extracellular matrix, cell adhesion molecule (Triulzi et al., 2013)
DEGs in Younger Women of the African Population	
CPB1 (Carboxypeptidase B1)	High expression in patients with ductal carcinoma *in situ*, correlates with better outcomes (Kothari et al., 2021)
WDR17 (WD Repeat Domain 17)	Overexpression correlates with higher metastatic ability in breast carcinomas (Lee et al., 2016)
GRIA4 (Glutamate Ionotropic Receptor AMPA Type Subunit 4)	Overexpressed in women with high-risk breast cancer (Marino et al., 2022)
CLDN8 (Claudin 8)	Overexpression correlates with higher androgen receptor expression and poor outcomes (Zhang et al., 2020)
PENK (Proenkephalin)	Overexpression suppresses the ERK/Fos oncogenic signaling pathway (Liu et al., 2021b)
ABCA8 (ATP Binding Cassette Subfamily A Member 8)	Overexpression inhibits breast cancer proliferation (Lv et al., 2022)
RELN (Reelin)	Serine Protease, reported to be crucial for neuronal migration and adhesion, in addition to the facilitation of myeloma cell proliferation (Qin et al., 2017)
THBS4 (Thrombospondin 4)	Overexpression leads to breast cancer invasion and metastasis (McCart Reed et al., 2013)
JCHAIN (Joining Chain Of Multimeric IgA and IgM)	Prognostic marker and indicator of tumor aggression in luminal breast cancer (Wang et al., 2020)
PRSS3 (Serine Protease 3)	Acts as an oncogene and promotes breast cancer progression (Qian et al., 2017)

### 3.7 Identified hub genes reveal a higher tendency of young patients of different populations to develop metastasis

Common differentially expressed genes in both datasets were imported to string to construct a protein-protein interaction (PPI) network. PPI enrichment value of genes overexpressed in young patients of the white population was 0.00285, with an average local clustering coefficient of 0.385. This cluster had 13 nodes and 4 edges. Gene ontology of proteins in this cluster were related epidermal growth factor receptor binding (AREG, EREG, and FAM83B), with the highest strength in this cluster (2.12) and a false discovery rate (FDR) of 0.0057. Other genes in the cluster were related to receptor regulator activity (CXCL13, IGSF1, IL20, FLT3, and FAM83B). The top 5 genes were calculated using CytoHubba, by which the top 5 nodes were ranked by degree. Upon Maximal Clique Centrality (MCC) ranking, FLT3, AREG, EREG, followed by IGSF1 and ARHGAP36 scored the highest, indicating the highest number of significant interactions in the network, thereby they were identified in our analysis as the hub genes of the white population. While AREG and EREG are reported to be ligands of the EGF receptor, AREG is an autocrine growth factor, and a mitogen of fibroblasts, and EREG is involved in vascular remodeling and a stimulator of cell proliferation. FLT3 is a receptor tyrosine kinase that promotes the phosphorylation of AKT1, and the activation of RAS, promoting the activation of the MAPK pathway. Pathway analysis of genes in this cluster revealed Constitutive Signaling by Aberrant PI3K in Cancer.

In the Asian population, there was no interaction found between the genes overexpressed in young patients of this population. However, PPI enrichment value of genes overexpressed in young patients of the African population was 0.026, with an average local clustering coefficient of 0.167. This cluster had 12 nodes and 2 edges. Top 5 genes were calculated using CytoHubba, by which the top 5 nodes were ranked by degree. Upon Maximal Clique Centrality (MCC) ranking, CPB1 scored the highest, followed by PENK and PRSS3. KEGG pathway analysis of this cluster revealed a neuroactive ligand-receptor interaction, which was reported previously to be upregulated in breast cancer patients prone to brain metastasis, as it has roles in the colonization of breast cancer metastases to the brain (Zhang et al., 2021).

### 3.8 Pathway analysis reveals transcriptional deregulation in the breast tumors of young patients in the white population

**FIGURE 13:**
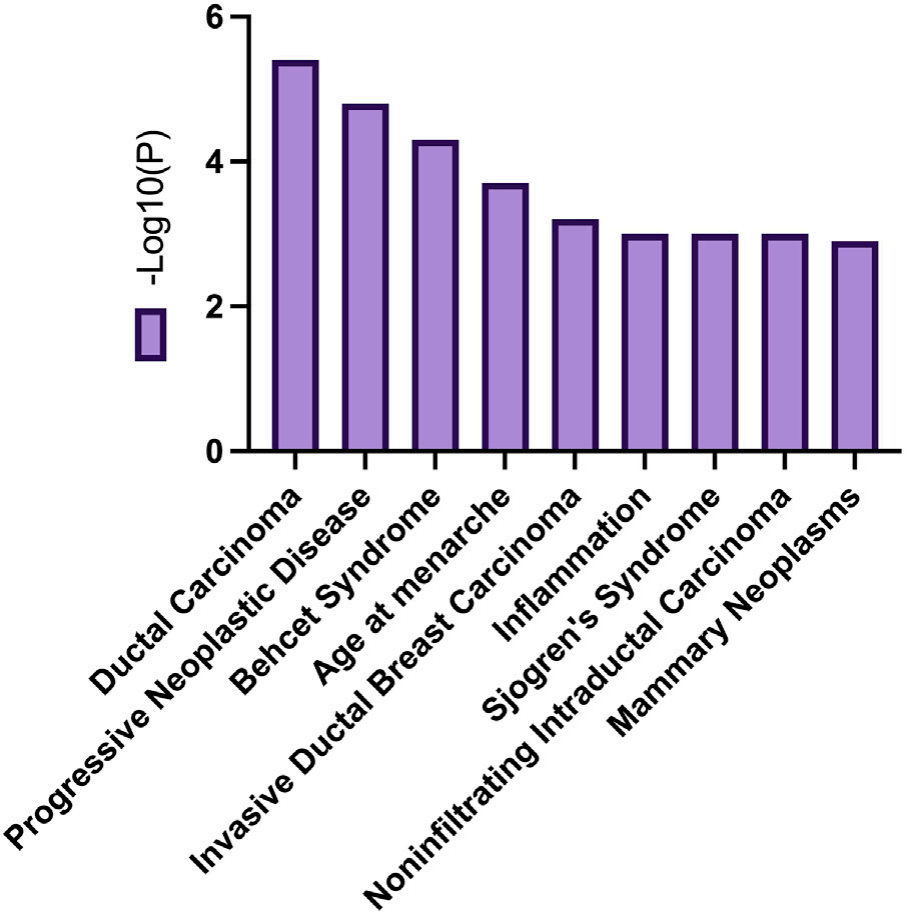
Common biological processes related to DEGs overexpressed in young women of the white populations.

The enrichment of differentially expressed genes of young patients from the white population in Metascape revealed that SP1 regulates the transcription of three genes in this cluster. KEGG pathway analysis of DEGs revealed the involvement of these genes in pathways related to ductal carcinoma, progressive neoplastic disease, Behcet Syndrome, age at menarche, and more illustrated in Figure 13. The pathways that were enriched for gene entries in young white patients were the MAP kinase pathway and PI3K/AKT pathway.

The enrichment of differentially expressed genes of young patients from the African population in Metascape did not reveal any common transcription factors regulating these genes. However, top-level Gene Ontology biological processes were multicellular organismal response to stress, Reelin pathway, and Neuroactive ligand-receptor interaction. Enrichment of DEGs overexpressed in the Asian population did not reveal any significant results.

## 4 Discussion

Research in recent years has focused on the identification of molecular mechanisms associated with cancer diagnosis, prognosis, and treatment, yet many cancer patients are still suffering from relapses, metastasis, and aggressive tumors. Around 24.5% of female cancer cases are breast cancer cases, making it the most commonly diagnosed cancer in women (Sung et al., 2021). Age has been a known risk factor for developing breast cancer, and in certain populations, breast cancer is being diagnosed a decade earlier, with more aggressive tumors and poorer prognosis. Patients with an early onset breast cancer (<40 years old) have the lowest survival outcomes compared to other age groups, which is suggested to be due to a higher grade of lymph node metastasis compared to other age groups (Lv et al., 2022). This is consistent with our study findings, by which young patients had the lowest disease-free survival compared to other age groups (Figure 5). However, the reasons behind the poor clinical outcomes of early onset breast cancer patients are hardly understood nor adequately studied. While most previously published studies focused on the genomics of early onset breast cancer in young patients of different populations, this study focused on using publicly available datasets in an attempt understand the transcriptomic profile underlying the aggressive nature of breast cancer in younger women in a population dependent manner. In our study, 35.14% of young African patients having basal tumors, compared to 19.35% of young white patients, by which it was found to be more prominent in older white women with breast cancer. These results are concordant with previous epidemiological studies, which can be explained by the delayed presentation to healthcare services among the African population compared to white women (Hirko et al., 2022). Our data suggests that even when young patients of different populations have a Luminal early onset breast cancer, they might have more aggressive tumors compared to older patients, as we found an overexpression of AREG, EREG, and DCX in younger patients of the white population who have Luminal or HER2+ tumors, compared to older patients of the same subtype, as the overexpression of these genes is associated with poor outcomes in breast cancer patients. On the other hand, genes that were overexpressed in the Asian population of the older age group indicate a higher metastatic ability of these tumors, suggesting higher grade tumors in older patients of the Asian population, compared to younger patients.

Our study sheds a light on the most significantly upregulated and amplified genes that might be associated with clinical outcomes of breast cancer in young women. Most genes that were found to be amplified in younger patients are involved in important signaling pathways that promote cancer progression and metastasis, such as AXIN2, which is involved in the activation of the Wnt/β-Catenin pathway, promoting the aggressiveness and invasive abilities of breast cancer cells (Azim et al., 2015). In addition, amplification of C17ORF58 is reported to target genes involved in the activation of the PI3K/Akt pathway and increases the metastatic potential of breast tumors. Interestingly, three long non-coding RNA genes were identified to be amplified in our analysis: LINC00332, LINC02875 and LINC00366. These long non-coding RNA genes have not previously been reported to be associated with breast cancer; however, Cheng M et al. reported that there is an association between the amplification of LINC02875 and the progression of glioma tumors (Cheng et al., 2021a). FREM2, which was found to be amplified in young patients in this study, was also reported to be associated with unfavorable outcomes in glioma patients (Zolotovskaia et al., 2021). These findings can possibly provide a new insight into the relationship between the amplification of FREM2 and LINC02865 and the development of glioma in young patients with breast cancer. Mezencev et al. reported an increased risk of glioma in young breast cancer patients and predicted a genetic contribution underlying the development of glioma in breast cancer patients below 45 years of age, which could be explained by the amplification of FREM2 and LINC02865 in young patients of our cohort (Mezencev, 2018).

In addition, we observed an enrichment of genes associated with Reelin pathway and Neuroactive ligand-receptor interaction in young African patients with breast cancer, which might indicate a crucial role in breast cancer metastases adapting to the brain microenvironment, thus indicating that these patients might be more prone to brain metastasis and disease progression. Previous studies have reported an enrichment of gene sets associated with tumor progression in Young African patients, which included mTOCRC1 and TGFß-signaling (Tonouo et al., 2022). However, our study is the first to propose an enrichment of the Reelin pathway gene sets in young African patients. On the other hand, White early onset breast cancer patients had an enrichment of genes related to the MAPK and PI3K/AKT pathways, which can indicate preferential hepatic colonization of breast metastases in those patients (Pierobon et al., 2017). Hub genes that were significantly expressed in young white patients were FLT3, AREG, EREG, IGSF1 and ARHGAP36. Enrichment of these genes in breast cancer could potentially indicate a higher metastatic ability of breast tumors, and activation of the PI3K/Akt pathway. In young African patients, CPB1, which correlates with better survival outcomes, was highly expressed. However, upon stratification by breast cancer subtype, there was hardly any expression of CPB1 in young TNBC African patients. In contrast, ART3 and AGT were highly expressed in young African patients with TNBC, compared to older patients. These genes are known to facilitate breast cancer migration, proliferation, and metastasis (Rodrigues-Ferreira et al., 2012; Tan et al., 2016).

Our study also proposed an association between Behcet’s syndrome and the development of early onset breast cancer in white young patients. While Behcet’s disease is frequently seen in Middle East, Japan and Mediterranean countries, there has been an association between this syndrome and the development of breast cancer and other malignancies (Kanazawa and Kammori, 2018).

This study provides strong evidence that the molecular profile of tumors derived from young breast cancer patients is can potentially explain the aggressiveness of these tumors on a population specific manner. However, our study suffered some limitations; firstly, the lack of some -omics in young breast cancer patients in the current literature that may be important for understanding the aggressive nature of breast cancer in a multiscale approach (lipidomics, metabolomics, epigenomics). Secondly, since other studies did not meet the inclusion criteria of our study, the main analysis that was performed to identify differentially expressed genes relied on data originating from a single study, and the majority of the patients were White, thereby the results of this study cannot be generalized on the populations studied. In addition, there were hardly any datasets that include multi-omic data of middle eastern breast cancer patients, where early onset breast cancer is a rising issue. Non-etheless, the findings of this study stress the need to conduct population-based multi-omic analyses to identify the potential drivers for tumorigenesis and molecular profiles of young breast cancer patients. This may provide valuable information for the management of patient outcomes and for developing a personalized, targeted therapeutics.

## 5 Conclusion

With all evidence given in this review, breast cancer in young women has a unique molecular profile. However, more research is needed in the Middle East to fully understand the molecular basis of breast cancer in young women, especially when it comes to the interactions and upregulated genes in the tumor microenvironment, since this can provide valuable information for the management of patients’ outcomes and to develop personalized, targeted approach to therapeutics.

## Data Availability

The datasets used to satisfy the aims of the study are included in the article/supplementary material and can be found in online repositories. Further inquiries regarding used datasets and databases can be directed to the corresponding author.
